# Comparisons of fentanyl and sufentanil on recovery time after inguinal hernia repair in children: a randomized clinical trial

**DOI:** 10.1186/s12893-024-02346-x

**Published:** 2024-02-14

**Authors:** Wen Chen, Hongyun Guoyang, Hui Yu, Yutong Xu

**Affiliations:** Department of Anesthesiology, Maternal and Child Health Hospital of Hubei Province, 745 Wuluo Road, Hongshan District, Wuhan, 430070 China

**Keywords:** Inguinal hernia, Fentanyl, Sufentanil, Pediatrics, Postoperative recovery time

## Abstract

**Background:**

Inguinal hernia repair is a common pediatric procedure. We studied postoperative recovery times in children undergoing laparoscopic inguinal hernia repair with anesthesia induced by fentanyl versus sufentanil.

**Methods:**

We performed a pilot randomized clinical trial between February and December 2022. Eligible children were assigned into two age groups, 2–6 and 6–12 years old groups. Then, children in each age group were randomly assigned into either the fentanyl (2 µg/kg) or sufentanil (0.2 µg/kg) group for anesthesia induction. Baseline characteristics were collected. The primary outcome was the postoperative recovery time, which was recorded as the time period from extubation to a Steward recovery score reaching 6. Secondary outcomes included surgical duration, anesthetic duration, intubation duration, and intraoperative hemorrhage.

**Results:**

There were 300 children, with 75 children in each group. In the 2–6 years old group, children who received fentanyl had statistically significantly shorter postoperative recovery times than children who received sufentanil (0.9 ± 0.4 versus 1.5 ± 0.3 h, *P* < 0.001). However, in the 6–12 years old group, children who received fentanyl had statistically significantly longer postoperative recovery times than children who received sufentanil (1.2 ± 0.4 versus 0.8 ± 0.4 h, *P* < 0.001). Baseline characteristics and secondary outcomes were comparable between two groups.

**Conclusions:**

Anesthesia induction with fentanyl or sufentanil resulted in different postoperative recovery times after laparoscopic inguinal hernia repair in children in different age groups. More studies are required to determine the appropriate induction anesthetic in children of different ages.

**Trial registration:**

The study protocol was retrospectively registered online at the Chinese Clinical Trial Registry (registration number ChiCTR2300072177, retrospectively registered on 06/06/2023).

## Background

The incidence of inguinal hernia was reported to range 1–5% in the pediatric population [1], with surgical repair of the hernia considered to be one of the most common surgical operations performed in children [2]. With advancements in minimally invasive surgery, the laparoscopic approach has become the most common procedure for repairing the inguinal hernia [3]. During the procedure, appropriate anesthesia is required to ensure adequate pain control and sedation. After the procedure, most children are sent to the post-anesthesia care unit (PACU), where they are closely monitored before being transferred to the medical ward or discharged home.

Patients should be fully awake with normal mental status before being discharged from the PACU. A delayed waking up period has been associated with more postoperative complications and prolonged length of hospital stay [4]. A prolonged stay in the PACU resulting in a shortage of beds could decrease the patient flow from the operating room to the PACU, slow down the surgical schedule, and increase patient dissatisfaction [5]. Certain medications, such as opioids, that are often used together with sedatives and muscle relaxants to induce anesthesia could result in delayed waking up in the PACU [6, 7]. Commonly used opioids include fentanyl and sufentanil. Fentanyl is a potent synthetic µ-receptor agonists [8]. Sufentanil is a fentanyl analogue but is more lipid-soluble and potent [9]. Most previous studies have shown effective analgesia of fentanyl and sufentanil in various surgical procedures, although sufentanil could show better hemodynamic stability, less respiratory depression, and fewer adverse effects [10, 11]. However, most studies were performed in adult patients, with little information on the effects of these opioids in children. Human brain development and metabolism change with age and childhood growth, which can affect opioid pharmacokinetics and pharmacodynamics [12, 13], which suggests that fentanyl and sufentanil might have different effects in children of different age groups, which has never been reported previously.

Therefore, we performed a pilot randomized controlled clinical trial and compared postoperative recovery times in children undergoing laparoscopic inguinal hernia repair under anesthesia induced by fentanyl versus sufentanil, with the aim to provide a reference for future research on the appropriate anesthetics to use in children of different age groups. We present our research in accordance with the CONSORT reporting guidelines [14].

## Materials and methods

### Study design and participant selection

We performed a randomized controlled clinical trial in pediatric patients who were scheduled for laparoscopic inguinal hernia repair at the Maternal and Child Health Hospital of Hubei Province in Wuhan, China, between February 2022 and December 2022. The study protocol was approved by the hospital ethics committee (approval number 2022-IEC113) and registered online at the Chinese Clinical Trial Registry (registration number ChiCTR2300072177, retrospectively registered on 06/06/2023). Informed consent was signed by the healthcare proxy of each child.

The inclusion criteria were pediatric patients who were (1) 2–12 years old; (2) scheduled for laparoscopic inguinal hernia repair; (3) classified as I–II according to the American Society of Anesthesiologists classification, and (4) tested as having normal preoperative hepatic and renal function. Excluded from the study were children with (1) chronic use of narcotics; (2) previous history of sleep disorders, obstructive sleep apnea, diabetes, epilepsy, mental illness, cardiovascular, cerebrovascular, or respiratory disease; (3) taking sedative and analgesic drugs 24 h before surgery; (4) diagnosed with speech, visual, or hearing disorders; (5) experiencing muscle strength difficulty in conducting the Steward score evaluation; or (6) participation in other clinical studies 3 months before the present study. In addition, those children with anesthesia duration < 80 min or > 200 min or intraoperative hemorrhage ≥ 80 mL were excluded from the final analysis.

### Baseline characteristics collection and group assignments

Baseline characteristics, including age, sex, body weight, and height, were recorded. Eligible children were assigned into two age groups, 2–6 years (2 years old ≤ age < 6 years old) and 6–12 years (6 years old ≤ age ≤ 12 years old) old groups. Then, each age group was randomly assigned into either the fentanyl or sufentanil group using a random number table. Finally, there were four groups in the present study, F1 (fentanyl, 2–6 years old), S1 (sufentanil, 2–6 years old), F2 (fentanyl, 6–12 years old), and S2 (sufentanil, 6–12 years old) groups.

### Study protocol

In the operating room, the child was placed under continuous monitoring of blood pressure, heart rate, respiratory rate, oxygen saturation (SpO_2_), and end-tidal carbon dioxide (ETCO_2_). In the fentanyl groups, general anesthesia was induced with propofol 2 mg/kg, cis-atracurium 0.15 mg/kg, and fentanyl 2 µg/kg. In the sufentanil groups, children received the same medications, except that fentanyl was replaced by sufentanil 0.2 µg/kg at the time of anesthesia induction (equianalgesic dosing fentanyl: sufentanil = 10:1 [15]). Children were intubated and connected to a ventilator with appropriate settings to keep the ETCO_2_ at 35–45 mmHg. During the procedure, all children were maintained under general anesthesia with sevoflurane, propofol, and remifentanil to keep changes of blood pressure and heart rate at no more than 25% of the baseline values. The body temperature was monitored through an oral thermometer. If the intraoperative temperature was out of the range of 36.2 to 37.3 °C, physical intervention, such as a warming fan or ice packs, was applied to ensure that the body temperature was within the normal range. The laparoscopic inguinal hernia repair was performed as described previously [1]. At the end of the procedure, sevoflurane, propofol, and remifentanil were terminated. Once the child had stable spontaneous respiration with SpO_2_ > 95% and EtCO_2_ < 50 mmHg, the endotracheal tube was removed, and the child was sent to the PACU for recovery.

### Outcome measurements

The primary outcome was the postoperative recovery time, which was the duration from the time point of extubation to the time point when a child had a Steward recovery score of 6. The Steward recovery score is a commonly used scale for determining recovery status [16]. It includes three dimensions, consciousness, respiratory tract patency, and autonomous activity, with a best score of 6 points [17].

Secondary outcomes included duration of surgery, anesthetic duration (from the initiation of anesthesia to the time point when a child regained full consciousness), length of intubation (from intubation to extubation), and intraoperative hemorrhage.

### Statistical analysis

The continuous data are presented as the mean ± standard deviation (M ± SD) or median with interquartile range (IQR), depending on the normality test results, and were compared by the paired *t*-test or Wilcoxon test, when appropriate. The categorical data are presented as numbers with percentages and were compared using the Chi square test. All statistical analyses were performed using SPSS software (version 13.0, IBM, New York, USA). A *P* < 0.05 was considered to be statistically significant.

## Results

### Participant enrollments and baseline characteristics comparisons

**Fig. 1:**
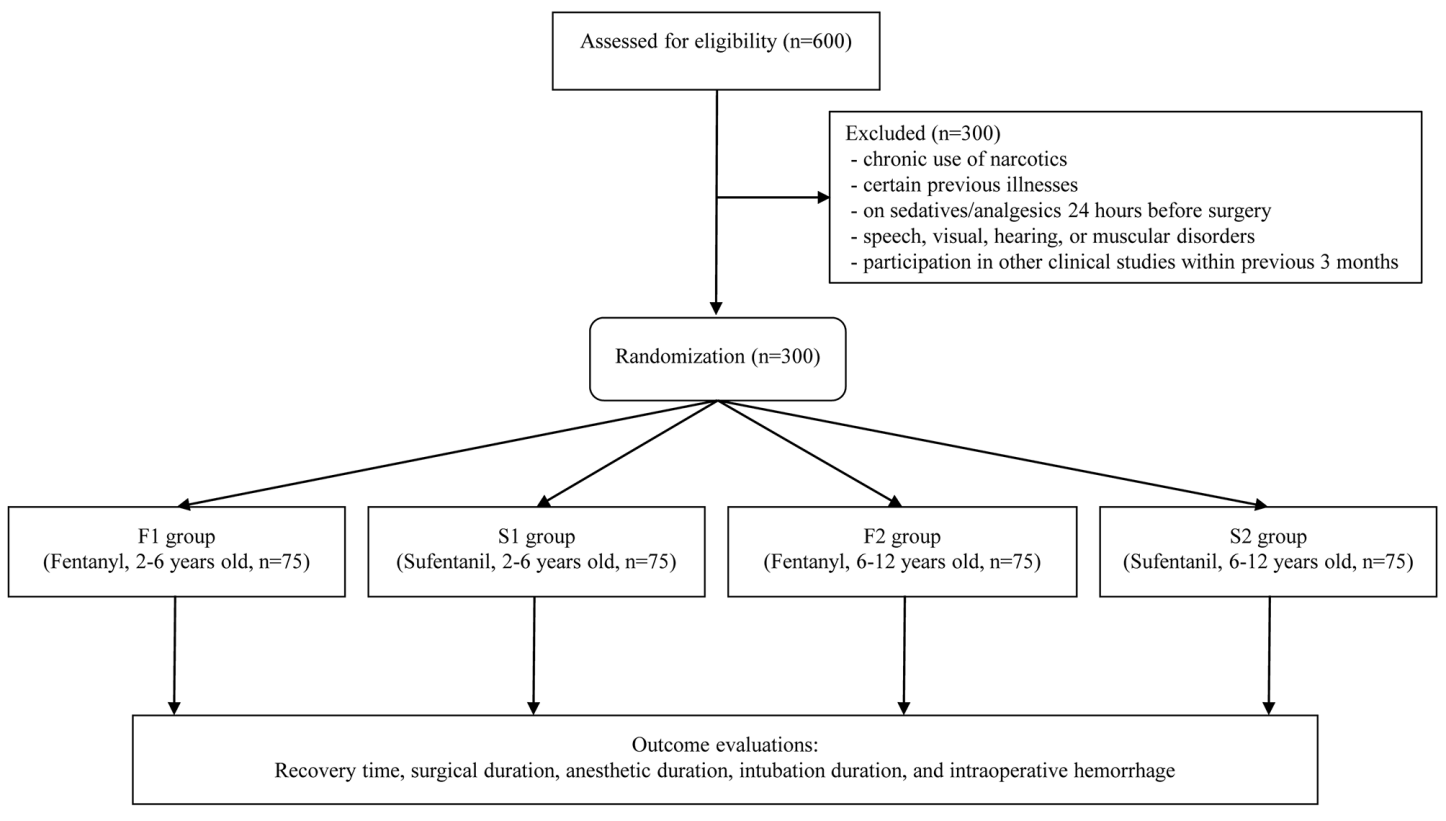
CONSORT flow diagram

A total of 300 children were included in the present study, with 75 children in each group (F1, F2, S1, and S2 groups). The CONSORT flow diagram is shown in Fig. 1. All children completed the study, with none of them excluded from the final analysis. The baseline characteristics comparisons showed comparable results between children in each age group and those under different treatments (Table 1). The dosages of fentanyl or sufentanil were 31.9 ± 4.7, 62.1 ± 12.3, 3.3 ± 0.5, and 6.0 ± 1.4 µg in the F1, F2, S1, and S2 groups, respectively.

**Table 1:** Baseline characteristics comparisons

Characteristics	F1 (*n* = 75)	S1 (*n* = 75)	*P*	F2 (*n* = 75)	S2 (*n* = 75)	*P*
Age, years, M ± SD	3.6$\pm$1.4	3.6$\pm$1.3	0.967	8.9$\pm$1.6	8.7$\pm$1.5	0.334
Sex, M, n (%)	40(53.3)	48(64.0)	0.185	44(58.7)	40(53.3)	0.511
Height, cm, M ± SD	102.0$\pm$9.8	103.3$\pm$8.6	0.423	133.5$\pm$9.7	130.8$\pm$9.4	0.089
Weight, kg, M ± SD	16.0$\pm$2.4	16.5$\pm$2.3	0.156	31.1$\pm$6.1	29.9$\pm$6.8	0.311

M ± SD, mean ± standard deviation; F1, fentanyl in children 2–6 years old; S1, sufentanil in children 2–6 years old; F2, fentanyl in children 6–12 years old; S2, sufentanil in children 6–12 years old

### Primary outcome comparisons

As shown in Table 2, in the 2–6 years old group, children who received fentanyl (F1 group) had statistically significantly shorter postoperative recovery times than children who received sufentanil (S1 group) (F1 versus S1, 0.9 ± 0.4 versus 1.5 ± 0.3 h, *P* < 0.001). In the 6–12 years old group, children who received fentanyl (F2 group) had statistically significantly longer postoperative recovery times than children who received sufentanil (S2 group) (F2 versus S2, 1.2 ± 0.4 versus 0.8 ± 0.4 h, *P* < 0.001).

**Table 2:** Comparisons of postoperative recovery time

Outcome	F1 (*n* = 75)	S1 (*n* = 75)	*P*	F2 (*n* = 75)	S2 (*n* = 75)	*P*
Recovery time, min mean ± standard deviation*	47.5 ± 17.4	74.1 ± 21.5	< 0.001	89.1 ± 19.9	46.8 ± 18.8	< 0.001

*, measured from the time point of extubation to the time point when the Steward recovery score reached 6; F1, fentanyl in children 2–6 years old; S1, sufentanil in children 2–6 years old; F2, fentanyl in children 6–12 years old; S2, sufentanil in children 6–12 years old

### Secondary outcome comparisons

The secondary outcome comparisons showed no statistically significant differences in the duration of surgery, anesthetic duration, length of intubation, or intraoperative hemorrhage between children in each age group and those under different treatments (Table 3).

**Table 3:** Secondary outcome comparisons

Outcomes*	F1 (*n* = 75)	S1 (*n* = 75)	*P*	F2 (*n* = 75)	S2 (*n* = 75)	*P*
Duration of surgery, min	100.0$\pm$13.2	99.3$\pm$13.4	0.756	99.2$\pm$12.1	101.6$\pm$13.0	0.206
Anesthetic duration, min	104.3$\pm$13.2	104.0$\pm$13.4	0.902	103.9$\pm$12.2	106.3$\pm$13.2	0.230
Length of intubation, min	102.0$\pm$13.1	101.8$\pm$13.2	0.899	101.7$\pm$12.2	103.9$\pm$13.2	0.289
Intraoperative hemorrhage, mL	13.4$\pm$5.8	11.9$\pm$5.2	0.113	9.9$\pm$4.7	10.6$\pm$5.1	0.328

* All data are presented as the mean ± standard deviation; F1, fentanyl in children 2–6 years old; S1, sufentanil in children 2–6 years old; F2, fentanyl in children 6–12 years old; S2, sufentanil in children 6–12 years old

## Discussion

Inguinal hernia repair is a common pediatric operation. Adequate sedation and analgesia are essential for a successful procedure. Postoperatively, short-term sedation and rapid recovery are desired to avoid complications and prolonged length of hospital stay [18]. The duration of postoperative sedation is closely related to the medications that are used during the surgical operation. Opioids are the common medications used during the intraoperative period. Opioids have both sedative and analgesic effects. Morphine is the prototype of opioids. Fentanyl and its derivatives, such as sufentanil, are more potent and have fewer side effects. Compared with fentanyl, sufentanil is more potent and has a shorter duration of action [10, 11]. However, there have been limited previous studies that compared the postoperative recovery times between fentanyl and sufentanil in the pediatric population. In the present pilot randomized clinical trial, we showed that fentanyl and sufentanil resulted in different postoperative recovery times in pediatric patients in different age groups.

The human brain undergoes rapid growth after birth, especially between the ages of 2 to 6 years old [19]. The total volume of the human brain begins to increase significantly from the age of 3 years old, reaching 85–90% of the size of the adult brain by the age of 6, after which the growth rate gradually slows down. After the age of 6 years old, a child’s brain development is basically mature, while physical and sexual development accelerate, with rapid body metabolism [20, 21]. Therefore, our present study selected two age groups of children (2–6 years old and 6–12 years old) as the research targets for exploring the potential different effects of analgesics on the postoperative recovery time in children of different ages.

Li et al. performed a randomized controlled clinical trial in 76 children with a mean age approximately 7 years old [22]. These children received either fentanyl or sufentanil for anesthesia induction before tonsillectomy and adenotomy. Postoperatively, children in the fentanyl group were less sedated and thus had more rapid recovery than children in the sufentanil group 2 h after surgery. In another randomized clinical trial in 60 children (mean age approximately 5 years old) who received congenital cardiac repair surgery, children who received fentanyl or sufentanil for anesthesia induction had a similar average time of awakening after surgery [23]. In our present study, we assigned children into different age groups (children ages 2–6 years old in one group and children ages 6–12 years old in the other group). Then, from each age group, we randomly assigned children into either the fentanyl group or sufentanil group. Our results showed different postoperative recovery times between these two treatments in the two different age groups. In the younger age group (2–6 years old), children who received sufentanil induction took a longer time to recover than children who received fentanyl induction. However, in the older age group (6–12 years old), children who received sufentanil induction took a shorter time to recover than children who received fentanyl induction. This age-dependent difference between anesthesia induction with fentanyl versus sufentanil was never reported previously. We consider that it might be caused by the different pharmacodynamics and pharmacokinetics of opioids in children of different ages.

Both fentanyl and sufentanil act on the µ-receptor to induce analgesic effects. Sufentanil is 5–10 times more potent and has a higher affinity to the µ-receptor than fentanyl. In addition, sufentanil is 8–10 time more lipid soluble than fentanyl [13, 24]. Younger children might have an immature blood–brain barrier to allow more sufentanil pass through to the central nervous system. Sufentanil also stays in the central nervous system longer due to its higher lipid solubility than fentanyl. These factors could result in a longer postoperative recovery time in young children treated by sufentanil versus fentanyl. However, the drug clearance changes with age. At approximately 12 years old, sufentanil clearance was almost double that of fentanyl (12–16 versus 7 mL/min/kg) [13], A faster drug clearance might explain the shorter recovery time for sufentanil than fentanyl in older children. The exact underlying mechanism of the different effects of fentanyl and sufentanil in children of different ages requires further studies. However, our study results suggested that, for the first time, pediatric physicians should consider different effects of fentanyl and sufentanil when treating children in different age groups. In addition to age, other factors, such as sex, body mass index, medications used during and after the operations, dosage of medications, and comorbidities, might confound the relationship between age and postoperative recovery. In the pediatric population, most medications are given based on the body weight. However, other methods that consider the drug clearance were proposed to more accurately determine the medication dosage [25]. Future studies should consider the potential influences from other factors.

Our secondary outcome evaluations included duration of surgery, anesthetic duration, length of intubation, and intraoperative hemorrhage. Previous studies had inconsistent results for these outcomes. For example, Prakanrattana et al. reported that the postoperative extubation time was significantly longer in the sufentanil group than in the fentanyl group in children after congenital cardiac repair surgery [23]. Wang et al. showed that the length of mechanical ventilation was shorter in the sufentanil group than in the fentanyl group in children after ventricular septal defect repair [26]. In the present study, we did not observe any statistically significant differences in these secondary outcomes between two treatments for both age groups. The varied results from different studies might be due to the different anesthetic regimen, surgical procedure, perioperative care, and methods used to calculate these secondary outcomes.

The strength of our pilot study was that it was the first study to compare fentanyl and sufentanil in children in different age groups. Our study found different postoperative effects for fentanyl compared to sufentanil in these children, which suggested that different analgesic medications should be considered in children of different ages. The limitations of our study were its small sample size in a single research center. We only studied the postoperative recovery time but did not investigate the pain intensity or potential side effects from the medications in this pilot study. Future randomized clinical trials should address these questions.

## Conclusions

In conclusion, anesthesia induction with fentanyl or sufentanil could result in different postoperative recovery times after laparoscopic inguinal hernia repair in children in different age groups. Fentanyl caused a short postoperative recovery time in children ages 2–6 years old, whereas sufentanil caused a short postoperative recovery time in children ages 6–12. More studies are warranted to investigate the different effects of fentanyl and sufentanil in children of different ages.

## Data Availability

Data are available from the corresponding author (2116628669@qq.com) upon request.

## Abbreviations

PACU: Post-anesthesia care unit
SpO2: Oxygen saturation
ETCO_2_: and End-tidal carbon dioxide
M ± SD: mean ± standard deviation
IQR: Interquartile range
